# COVID-19’s impact on worker stress in human service organizations: The mediating role of inclusion

**DOI:** 10.1371/journal.pone.0295743

**Published:** 2023-12-11

**Authors:** Magdalena Calderón-Orellana, Andrés Aparicio, Nicolás López–Huenante

**Affiliations:** 1 School of Social Work, Pontificia Universidad Católica de Chile, Santiago, Chile; 2 Millennium Institute for Care Research (MICARE), Santiago, Chile; 3 Facultad de Administración y Economía, Universidad de Santiago, Santiago, Chile; 4 Master in Applied Economics, Pontificia Universidad Católica de Chile, Santiago, Chile; Pontificia Universidad Catolica de Chile (PUC) / Universidad de Valladolid (UVa), SPAIN

## Abstract

Human service organizations faced extraordinary challenges due to COVID-19. Despite the increasing interest and research in this new scenario, there has been limited discussion about the impact of COVID-19 on workers, the challenges they faced, and the resulting stress. This study aimed to analyze the impact of COVID-19 on work-related stress and the mediating role of inclusion among workers in human service organizations in Chile during the pandemic. The research design was quantitative and involved a sample of 173 workers from civil society organizations who were contacted during the pandemic. The study confirmed that individuals most affected by the pandemic experienced higher levels of work-related stress, and that inclusion played a negative mediating role in this relationship. This article highlights the importance of relationships, decision-making processes, and access to information in reducing stress in post-COVID scenarios for organizations that traditionally handle crises.

## Introduction

During the COVID-19 pandemic, the well-being of workers in human service organizations was threatened by an unprecedented scenario that forced them to adapt their methods of organization, planning, fundraising, and work [1, 2]. Despite its relevance in relation to the impact on the services they provide, the well-being and quality of life of workers in human service organizations have received limited attention compared to the healthcare sector. Indeed, research has shown that healthcare workers have experienced stress and anxiety [3–5], post-traumatic stress disorder (PTSD) [6, 7], psychological distress [8], and burnout [9, 10]. However, when it comes to social service workers, the information available is limited.

Certainly, as a result of isolation and quarantine measures, human and social services have required a reevaluation, introducing new adaptations and challenges for civil society organizations [11]. Workforce reductions and the discontinuation of certain services compelled workers to confront substantial increases in their workloads [12], adding further strain to already overburdened services and employees [13]. Moreover, the heightened expectations from citizens regarding social services have created difficulties for these workers in balancing organizational requirements, citizen requests, and professional values [14].

Despite the clear connection between the COVID-19 catastrophe and the job-related challenges stemming from organizational adaptations, there is limited evidence on the stress experienced by workers in human service organizations due to COVID-19. This is significant considering the pivotal role of stress in job satisfaction, turnover within these organizations [15], and its impact on service quality. Indeed, threats to the well-being of social service workers can pose risks to the entire social protection system [16, 17].

Therefore, this research aims to determine the impact of COVID-19 on the stress experienced by workers in nonprofit human service organizations.

The study of workers’ stress in nonprofit human service organizations and social services has taken on special relevance since the 1990s [18] because of the impact of stress on workers’ well-being and performance. Studies have revealed that high work pressures, a lack of control over decision-making, ethical dilemmas in the workplace, the low inclusion, low pay, and inadequate supervision, among other factors, have been identified as sources of stress in these workers [16, 19–22]. Recognizing that low perception of inclusion, understood as the individual’s sense of being part of the organization in formal and informal processes [23], has been associated to workers’ stress [15, 24] and that the pandemic has affected ways of organizing work and of relating, it seems relevant to ask about the role of inclusion in the relationship between COVID-19’s impact and stress. In other words, it is crucial to consider the value of inclusion in mitigating stress in a context of crisis, where organizations and workplace relationships have undergone significant changes.

Thus, this research examined the role of inclusion in the relationship between COVID-19’s impact and stress and assessed if this relationship was maintained in workers at nonprofit organizations in Chile during the peak of the disease in 2021. This study is of significant relevance as it provides insights into a group that faced high demand during the pandemic. Moreover, it contributes to better understand a phenomenon that must be addressed to reduce the effects of stress on health [8, 25–27]. Additionally, it sheds light on the challenges experienced by organizations, enabling the development of strategies to cope with stress. Given the connection between the effectiveness of such services and worker well-being, these strategies can enhance the well-being of both workers and users of social services [17]. Furthermore, there is a growing demand for research on nonprofit human service organizations that seeks to clarify how to address these challenges. Finally, in considering the experience of Chile, this research adds to the study of the reality of civil society organizations from a particular perspective that until now has been underexplored [28]. In doing so, it contributes to building an understanding of stress in these organizations, complementing existing knowledge.

### Well-being and stress in social service workers during pandemic of COVID-19

The scarcity of evidence related to the impact of the COVID-19 health emergency on the stress levels of nonprofit human services workers is noteworthy. Since the onset of the pandemic in March 2020, when the World Health Organization declared the disease caused by the SARS-CoV-2 virus a pandemic, most studies have focused on assessing the presence of stress in the field of social services, with inadequate attention to the effects of COVID-19 on stress.

As such, studies conducted to date have produced data that highlight elevated levels of stress in this group of workers. For instance, the study by Martínez-López et al. [29] identified symptoms of burnout in social services personnel during the initial wave of the pandemic in Spain, with high rates of emotional exhaustion (53.8%) and depersonalization (35.1%). In a similar vein, Dima [30] reported that stress is more strongly associated with work-related burnout than with depersonalization.

Furthermore, a study involving 181 workers engaged in social services during the COVID-19 pandemic has shown that, despite expressing significant job satisfaction rooted in compassion, these professionals exhibited symptoms of post-traumatic stress disorder, burnout, and secondary trauma [31]. In other words, these individuals experience prominent levels of stress while remaining committed to their work.

Additionally, stress has been linked to depression, anxiety, and organizational support, and has been found to have a negative correlation with resilience [32]. From a broader perspective, Alsabti [33] examined the social, familial, and economic stress experienced by 135 workers from 22 social service centers serving individuals with disabilities in Saudi Arabia. This study revealed a significant relationship between social stress and burnout, as well as an inverse correlation with personal achievement.

In summary, the available evidence underscores that nonprofit human services workers have faced high levels of stress during the COVID-19 pandemic, resulting in symptoms of burnout, post-traumatic stress disorder, and other mental health issues.

Numerous studies have explored the impact of COVID-19 on the quality of life in various populations. While limited evidence specifically pertains to social service workers, research on the general population sheds light on the broader effects of the pandemic on well-being.

In the Philippines, a study focused on Filipino teachers found a significant difference in the impact of COVID-19 on quality of life based on education level. However, factors such as age, gender, marital status, employment status, income, proximity to COVID-19 cases, personal connections to COVID-19 patients, presence of underlying medical conditions, and perceived threat did not significantly affect the quality of life [34]. Another study in the Philippines involving nursing students, reported that the pandemic had a moderate impact on their quality of life. This impact varied considerably based on gender and proximity to COVID-19 cases. Furthermore, the study revealed a moderate inverse relationship between psychological resilience and the impact of COVID-19 on quality of life [35].

Khodami and colleagues conducted a global study involving 3,002 participants and found that quality of life had significantly deteriorated over time as the pandemic progressed. Perceived stress levels had risen, and emotional regulation became more challenging. Prolonged quarantine durations were associated with worsening quality of life, increased perceived stress, and greater difficulties in emotional regulation [36].

In Saudi Arabia, research by Islam and Alharthi involving 506 households confirmed a significant reduction in the quality of life due to the COVID-19 crisis. Specific factors like low income, large household size, male-headed households, urban residence, household head unemployment, low educational levels, and the presence of elderly members were associated with a higher impact of COVID-19 on quality of life [37].

Singaporean researchers observed a direct correlation between COVID-19 stress syndrome and a decline in quality of life. This stress syndrome was inversely related to feelings of gratitude [38]. Meanwhile, a study conducted by Maric and colleagues [39], with a sample of 251 patients, indicated that the pandemic’s impact on quality of life exceeded the theoretical average on a 5-point scale. Surprisingly, there was no significant association between COVID-19’s impact and demographic characteristics or patient diagnoses, highlighting that demographic factors did not affect the pandemic’s influence on quality of life.

While the specific effects on social service workers remain an area for further investigation, these studies collectively emphasize the broad-reaching impact of the pandemic on the quality of life in different populations and underscore the importance of understanding the multifaceted aspects of well-being during such crises.

From these numerous studies, it is possible to establish, on the one hand, that social service workers experienced stress during the pandemic. On the other hand, COVID-19’s impact on quality of life did not necessarily depend on some demographic aspects but eventually on external effects such as the time of exposure to the pandemic and sustained demands over time. That is, it was related to the workers’ job demands, which could be explained from the perspective of job demands-resources theory [40].

Job demands refer to those physical, psychological, organizational, or social aspects of work that require sustained effort and have physiological and psychological effects. Work resources refer to the physical, psychological, organizational, or social aspects of work that reduce the demands at work, achieve the objectives of the work, and stimulate personal development [41]. Resources allow for the prediction of outcomes such as satisfaction, motivation, and engagement [42].

According to this theory, demands and resources trigger independent processes linked to people’s health and motivation. Demands are predictors of stress, and resources are predictors of job satisfaction, well-being, and engagement. However, although demands and resources initiate independent processes, they can also have combined effects. Given the basic precepts of this theory, it can be said that COVID-19’s impact on quality of life is a demand for workers and therefore affects stress. This is how the first hypothesis of our study is configured:

*H1*: *COVID-19’s impact on quality of life has a direct and positive relationship with the stress of nonprofit organizations workers*, *both in its dimension of exhaustion and disengagement at work*.

### Inclusion processes and stress in catastrophe and disaster situations

Considerable evidence supports the positive impact of inclusion on both individuals and organizations under typical circumstances [24, 43–45]. Yet, research addressing the role of inclusion during times of crisis remains limited.

Chan et al. [46]examined how company characteristics influence inclusion practices for employing individuals with disabilities. Their findings underscored the critical role of company size in shaping inclusion practices, with smaller firms less likely to implement such practices. Parker et al. [47] explored workplace inequalities in the public service sector and revealed that approaches rooted in liberal or radical "soft" equality discourses led to gradual, work-focused improvements, particularly for women.

Ahmad et al. [48] identified a positive relationship between spiritual leadership and organizational inclusion practices, although the study found that the Islamic ethics of a doctor did not significantly impact organizational inclusion practices. In Thailand, Rodprayoon and Maj [49] reported that diversity and inclusion in the workplace had a more substantial effect on employee retention during COVID-19 than perceived organizational support.

Kuknor and Bhattacharya [50] established a positive association between organizational inclusion and organizational citizenship behavior, as well as organization-based self-esteem. This highlights the importance of actively managing workplace inclusion for positive organizational outcomes.

However, as with stress, although studies reported evidence regarding inclusion during the pandemic, evidence of the effect of the pandemic on inclusion was not found.

It has been established that inclusion, and particularly the perception of inclusion, is associated with organizational processes, including both formal and informal participation, access to information, and decision-making [23]. Consequently, it is plausible to assert that changes in these processes—participation, information, and decision-making—can impact the perception of inclusion. Furthermore, if we recognize that the pandemic affected the ways in which employees interact and make decisions due to COVID-19, it is reasonable to contend that individuals affected by COVID-19 may also have experienced changes in their levels of inclusion. This leads to the proposition of our second hypothesis:

*H2*. *The COVID-19’s impact will be negatively and directly related to the perception of inclusion of nonprofit organization workers*.

In a similar vein, assuming that inclusion signifies access to resources highly valued by an organization [51], one can contend, from the perspective of the Job Demands-Resources theory, that inclusion plays a role in stress. This linkage between inclusion and stress has indeed been identified by Hopkins et al. [52]in their study on factors contributing to turnover among childcare workers, revealing a correlation between inclusion, stress, and worker turnover. Moreover, the association between exclusion and stress among employees in an American company has also been investigated [53], as well as the connection between inclusion in decision-making processes and role conflict [54]. Building on this foundation, we propose the following hypothesis:

*H3*. *The perception of inclusion will be negatively and directly related to stress in nonprofit organization workers*.

Finally, considering the relationship between the effect of COVID-19 and perception of inclusion and the relationship of this with stress, we can establish the following hypothesis:

*H4*. *COVID-19’s impact on quality of life will indirectly relate to stress through the perception of inclusion among nonprofit organization workers*.

**Fig 1:**
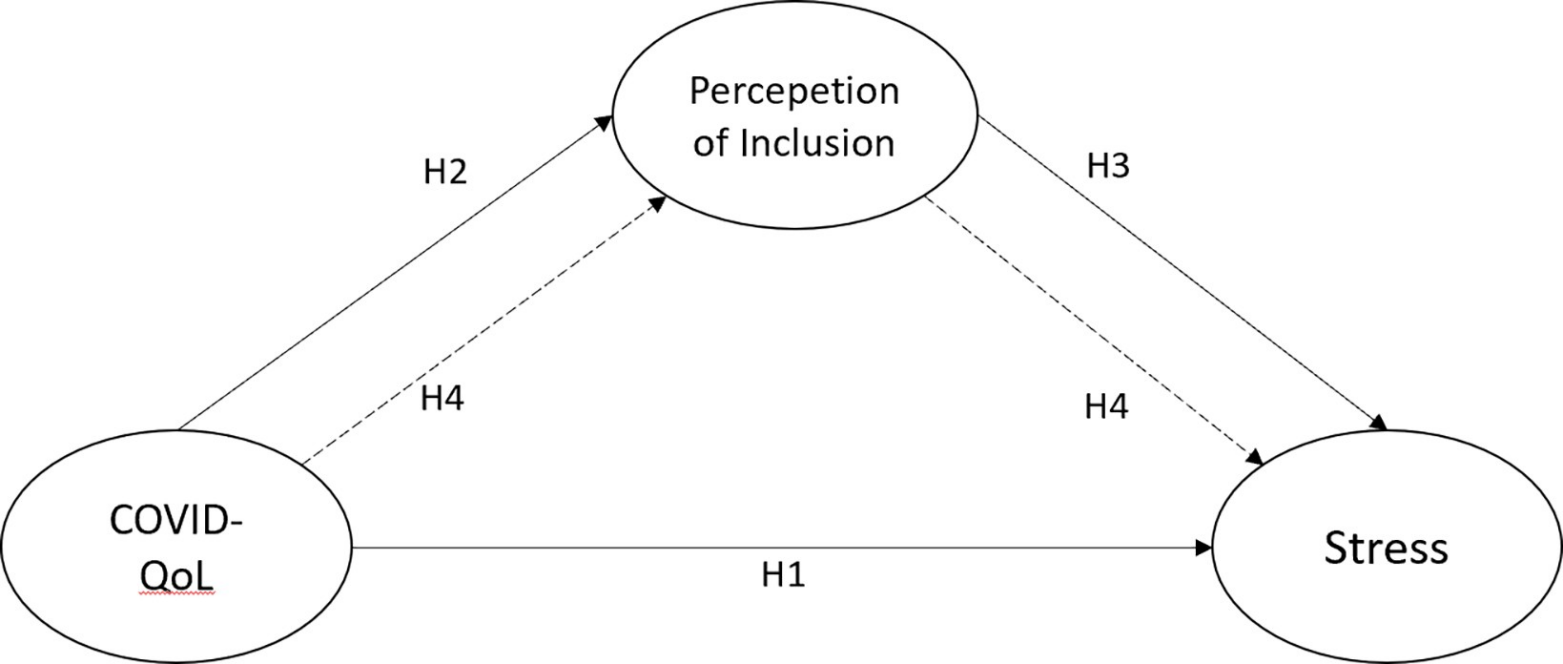
Hypothetical model.

Fig 1 presents the hypotheses and the hypothetical model.

## Methodology

To assess the hypotheses of this study, an exploratory quantitative observational research design was employed.

### Sample

The research targeted workers employed by nonprofit civil society organizations in Chile that were dedicated to social objectives such as poverty alleviation, inclusion of individuals with disabilities, development of historically marginalized areas, and the facilitation of access to social rights. Initially, contact was established with the human resources units of six organizations. To encourage participation, each organization was promised access to aggregated results information.

Ultimately, four organizations, each with a presence in different cities across Chile, agreed to participate in the study. The sampling method employed was non-probability and convenience sampling. Initially, the message was sent to 499 individuals, resulting in 242 responses at time point 1 and 200 responses at time point 2. A total of 173 individuals completed both surveys, primarily consisting of well-educated women from medium to high socioeconomic backgrounds, as well as young individuals.

Through the Vice-Rectory of Public Engagement of the University of Santiago de Chile, contact was made with the organizations’ human resources units. The sample recruitment began in June. The sample was accessed via email sent by the human resources unit of each organization or by the responsible researcher during June and September of 2021. The investigation team conducted the detailed content and wording of the message. Everyone was informed in detail of the objective of the study, its stages, the treatment of the information, and its anonymity and confidentiality.

### Measures

#### COVID-19’s impact

In order to assess the impact of the pandemic on quality of life, the COV19-QoL scale was used [55]. The scale evaluates the influence of COVID-19 on quality of life and observes the individual’s perceptions regarding the quality of life, its effects on physical and mental health, depression, and personal safety. To adapt the questionnaire for use in a Spanish-speaking population, the questionnaire, composed of six statements, was translated into Spanish and then assessed by three expert judges. The questionnaire is presented in Spanish and English in Table 1.

**Table 1:** COV19-QoL scale.

*Debido al CORONAVIRUS*:	*Due to the spread of the coronavirus*
Creo que mi calidad de vida es peor que antes	My quality of life is lower than before
Creo que mi salud mental se ha deteriorado	I think my mental health has deteriorated.
Creo que mi salud física se ha deteriorado	I think my physical health may deteriorate
Me siento más tensa(o) que antes	I feel more tense than before
Me siento más deprimida(o) que antes	I feel more depressed than before
Siento que mi seguridad personal está en riesgo	My personal safety is at risk

The measure was applied using a Likert-type questionnaire where individuals were presented with statements and asked to identify their level of agreement with each sentence ranging from 1 (strongly disagree) to 5 (strongly agree). The total score, which could be between 5 and 30, was the sum of item scores, and higher scores indicated a more severe impact of COVID-19 on quality of life.

Reliability was assessed with Cronbach’s alpha, reaching 0.89, indicating strong internal consistency. Validity was evaluated through Confirmatory Factor Analysis (CFA), which revealed a good fit (RMSEA = 0.08, 90% CI [0.039, 0.13]; CFI = 0.98; TLI = 0.97; SRMR = 0.03), affirming the questionnaire’s ability to measure the intended constructs effectively.

#### Stress

The Oldenburg Burnout Inventory (OLBI) was employed to assess stress [56]. This instrument measures a worker’s stress level and consists of two subscales. The exhaustion subscale measures the outcomes of intense physical, emotional, and cognitive stress resulting from prolonged exposure to specific job demands. The second subscale, disengagement from work, pertains to the individual’s sense of detachment from work in a general sense, as well as from the work’s objectives and content. In addition, attachment items refer to the relationship between an employee, their job position, and their identification with the job. The scale has 16 questions and was presented as a Likert-type questionnaire scale of 1 to 5, where 1 is “strongly agree,” and 5 is “strongly disagree.” Due to the instrument consisted of positive and negative statements, the latter had to be calculated in reverse (R). The final score of the scale and subscales could be between 1 and 5, with the highest scores indicating the highest stress level. The scale in Spanish and English are presented in Table 2.

**Table 2:** OLBI scale.

*Spanish version*	*English version*
Encuentro, con frecuencia, aspectos nuevos e interesantes en mi trabajo.	I always find new and interesting aspects in my work.
Hay días en que me siento cansado antes de llegar al trabajo (R)	There are days when I feel tired before I arrive at work (R)
Cada vez hablo, con más y más frecuencia, sobre mi trabajo de manera negativa (R)	It happens more and more often that I talk about my work in a negative way (R)
Después de trabajar, necesito más tiempo que antes para relajarme y sentirme mejor (R)	After work, I tend to need more time than in the past in order to relax and feel better(R)
Puedo tolerar muy bien la presión de mi trabajo	I can tolerate the pressure of my work very well.
Últimamente, suelo pensar menos en mi trabajo y hago mis tareas laborales casi mecánicamente (R)	Lately, I tend to think less at work and do my job almost mechanically (R)
Considero que mi trabajo es un desafío positivo	I find my work to be a positive challenge.
Durante mi trabajo, me siento emocionalmente agotado (R)	During my work, I often feel emotionally drained (R)
Con el tiempo, uno puede desconectarse de este tipo de trabajo (R)	Over time, one can become disconnected from this type of work (R).
Después de trabajar, tengo suficiente energía para mis actividades de ocio.	After working, I have enough energy for my leisure activities.
A veces me siento agotado por mis tareas de trabajo (R)	Sometimes I feel sickened by my work tasks (R)
Después de mi trabajo, normalmente me siento cansado y sin energía (R)	After my work, I usually feel worn out and weary (R)
Este es el único tipo de trabajo que me imagino haciendo (R)	This is the only type of work that I can imagine myself doing (R)
Por lo general, puedo manejar bien la cantidad de trabajo que tengo	Usually, I can manage the amount of my work well
Me siento cada vez más comprometido con mi trabajo	I feel more and more engaged in my work.
Cuando trabajo, normalmente me siento con energía	When I work, I usually feel energized.

Reliability reached 0.84, measured with Cronbach’s alpha. However, the result for validity through CFA did not show an adequate fit. Specifically, question 9 exhibited a negative relationship with the other questions in this sample. After item 9 was removed, the scale exhibited a good fit (RMSEA = 0.09, 90% CI [0.083, 0.11]; CFI = 0.84; TLI = 0.81; SRMR = 0.08).

#### Inclusion

The Mor Barack perception of inclusion scale was used as a Likert-type questionnaire [23]. The scale comprises Likert-type indicators of 1–5 to evaluate the level of agreement (1, “strongly disagree” and 5, “strongly agree.”) Higher scores indicated a greater sense of inclusion. As this instrument consisted of positive and negative statements, the latter had to be calculated in reverse (R).

The application of the instrument did not include the questions regarding participation in informal spaces, as it was conducted during the COVID-19 pandemic when informal participation scenarios were limited. In this way, each subscale—organization, supervisor, team, and higher management inclusion—included three items, which were added, giving the possibility of scoring between 3 and 15 in each subscale and 60 on the scale. The scale, already validated in Spanish [57], had good reliability (0.85). Additionally, it exhibited an adequate fit with values (RMSEA = 0.07, 90% CI [0.04, 0.09]; CFI = 0.94; TLI = 0.92; SRMR = 0.05). The Spanish and English versions of the scales are presented in Table 3.

**Table 3:** Perception of inclusion scale.

*Spanish version*	*English version–original version*
Tengo influencia en las decisiones tomadas por mi grupo de trabajo respecto a nuestras tareas	I have influence in decisions taken by my work group regarding our tasks.
Mi equipo comparte abiertamente información conmigo relacionada al trabajo	My coworkers openly share work related information with me
Generalmente, estoy considerado/a e invitado/a a participar en actividades relacionadas con mi grupo de trabajo	I am typically involved and invited to actively participate in work related activities of my work group
Soy capaz de influir sobre las decisiones que afectan a la organización	I am able to influence decisions that affect my organization
Generalmente, soy de los/as últimos/as en enterarme de los cambios importantes en la organización (R)	I am usually among the last to know about important changes in the organization(R)
Generalmente, estoy invitado/a a reuniones importantes en mi organización	I am usually invited to important meetings in my organization
Frecuentemente, mi jefatura directa pide mi opinión antes de tomar decisiones importantes	My supervisor often asks for my opinion before making important decisions.
Mi jefatura directa no comparte información conmigo (R)	My supervisor does not share information with me (R)
Mi jefatura directa me invita a participar activamente de reuniones de revisión y evaluación de mi trabajo	I am invited to actively participate in review and evaluation meetings with my supervisor.
Generalmente, me invitan a dar mi opinión en reuniones con jefaturas superiores a mi jefatura directa	I am often invited to contribute my opinion in meetings with management higher than my immediate supervisor.
Frecuentemente, recibo información de gerencias o de cargos más altos que mi jefatura directa (Ej. correos electrónicos)	I frequently receive communication from management higher than my immediate supervisor (i.e., memos, emails)
Generalmente, me invitan a participar de reuniones con gerencias o cargos más altos que mi jefatura directa	I am often invited to participate in meetings with management higher than my immediate supervisor.

#### Control variables

Information regarding the age, nationality, experience, education and occupation, and SES of participants was collected for control purposes.

### Procedure

The study included the application of two online questionnaires through Qualtrics© software. The first questionnaire collected demographic information, while the second, administered 2 to 4 weeks later, observed the scales related to the impact of COVID-19, stress, and inclusion. The first questionnaire was answered by 242 individuals, while the second one was completed by 200 participants. 173 individuals responded to both assessments. Participants were contacted via email, and reminders were sent during the two weeks when the questionnaire remained open. Access to the sample was coordinated with the human resources units of each organization, ensuring that it did not interfere the organization’s operations.

Before data collection, official written ethical approval was obtained from the Research Ethics Committee of the Universidad de Santiago de Chile (Approval Number: 222/2021) on June 18, 2021. Participants were informed about the purpose of the study, the content of the questionnaires, confidentiality, and the fact their participation was anonymous and voluntary. They were requested to provide a written, online recorded informed consent before completing the survey.

Data collection commenced in June and ended in October 2021. The database was created in the statistical software SPSS 25. Subsequently, tests pertaining to the model and hypotheses, including correlation studies, multiple linear regressions, and structural equations, were conducted using Mplus version 7. The measurement variables within the hypothetical model did not exhibit missing values.

## Results

The details of its characteristics can be seen in Table 4.

**Table 4:** Characteristics of the sample.

*Variables*	*n*	*%*	*Min*	*Max*	*Mean*	*SD*
*Gender*	173					
Men	48	27.7%				
Women	125	72.3%				
Missing values	27					
*Age (years)*			24	65	38.79	9.88
*Educational level*	173					
Primary School	0	0%				
Secondary School	8	4.6%				
Technical Education	17	9.8%				
Professional Education	80	43.2%				
Postgraduate	68	39.3%				
Missing values	27					
*Socioeconomic status*	170					
1 (poor people)	0	0%				
2 (vulnerable people)	6	3.5%				
3 (lower middle class)	16	9.4%				
4 (typical middle class)	18	10.6%				
5 (emerging middle class)	48	28.2%				
6 (affluent middle class)	74	43.6%				
7 (high class)	8	4.7%				
Missing values	30					

These findings align with studies conducted on populations employed in child social-care services in Chile as well as in various international contexts, including China, the USA, and several European countries [16, 58, 59].

Table 5 presents descriptive statistics of the study variables and their correlation.

**Table 5:** Correlation results.

* *	*Mean*	*SD*	*1*	*2*	*3*	*4*	*5*	*6*	*7*
***(1) Tenure***	7.08	5.86	1						
***(2) Age***	38.79	9.88	0.67**	1					
***(3) Educational level***	4.2	0.79	-0.22**	-0.3**	1				
***(4) SES***	5.13	1.19	-0.05	-0.13	0.63**	1			
***(5) COVID-19’s impact***	2.94	0.94	-0.11	-0.17*	0.16*	0.18*	1		
***(6) Inclusion***	39.56	6.96	0.04	-0.05	0.15*	0.16*	-0.17*	1	
***(7) Stress***	2.66	0.59	-0.2**	-0.39**	0.19*	0.14	0.62**	-0.35**	1

*p < .05

**p < .01

According to the correlation matrix, COVID-19’s impact had a positive relationship with educational level, SES, and stress. It was also possible to observe that COVID-19’s impact was negatively related to age and inclusion. Likewise, stress had a negative relationship with age, tenure, and inclusion, and a positive relationship with educational level. Finally, inclusion is related to an individual’s educational level and SES.

Multiple linear regressions were performed to evaluate the hypotheses under study to evaluate each of the established relationships. The results are presented in Table 6.

**Table 6:** Hypothetical model regressions.

*Model*	*B*	*SE*	*RMSEA*	*90% CI RMSEA*	*CFI*	*TLI*	*SRMR*
*1*. *COVID-19 Impact predicts stress*:			0.08	0.066/0.086	0.87	0.86	0.08
Disengagement	0.52***	0.07					
Exhaustion	0.74***	0.04					
*2*. *COVID-19 Impact predicts inclusion*:			0.06	0.048/0.075	0.93	0.91	0.06
Working Group Inclusion	-0.2**	0.08					
Organization Inclusion	-0.25***	0.09					
Supervisor Inclusion	-0.08	0.1					
Higher Management Inclusion	-0.08	0.09					
*3*. *Inclusion predicts stress*:			0.07	0.059/0.076	0.84	0.82	0.08
Disengagement							
Working Group Inclusion	0.05	0.23					
Organization Inclusion	-0.83***	0.3					
Supervisor Inclusion	-0.08	0.17					
Higher Management Inclusion	0.32	0.21					
Exhaustion							
Working Group Inclusion	-0.03	0.22					
Organization Inclusion	-0.39	0.29					
Supervisor Inclusion	-0.04	0.18					
Higher Management Inclusion	0.22	0.20					

*p < .05

**p < .01

***p < .001

According to the results, COVID-19’s impact had a positive and direct relationship with stress through the dimension of exhaustion (β = 0.52, p < 0.001) and disengagement (β = 0.74, p < 0.001). Likewise, a direct and negative relationship was observed between COVID-19’s impact and inclusion, particularly through working group inclusion (β = -0.2, p < 0.01) and with organization inclusion (β = -0.25, p < 0.001). Finally, the third regression of the model confirmed that inclusion was negatively related to stress, particularly through organization inclusion and its relationship with disengagement from work (β = -0.83, p < 0.001).

The regression results indicated that COVID-19’s impact affected stress and the perception of inclusion. Inclusion likewise had effects on stress, and particularly disengagement. Therefore, the hypothetical model was evaluated through a structural equation model to advance in testing the hypotheses. The hypothetical model did not have a good fit (RMSEA 0.08, 90% CI [0.0.76, 0.089]; CFI 0.79; TLI 0.77; SRME 0.12).

However, considering the results of the regressions, we assessed two new models. The first considered the effect of COVID-19 on organization inclusion, as it seemed to be the one that presented meaningful results in this context. The second one was the same but only considered the subdimension disengagement in stress. In all cases, gender, age, tenure, SES, and race were controlled for. The results are shown in Table 7.

**Table 7:** Testing modified models.

*Model*	*RMSEA*	*90% CI RMSEA*	*CFI*	*TLI*	*SRMR*
1. Considering COVID-19 impact, stress and organization inclusion	0.08	0.069/0.086	0.84	0.82	0.09
2. Considering COVID-19 impact, stress–disengagement and organization inclusion	0.07	0.060/0.085	0.89	0.87	0.08

Based on the adjustment indices of the second model, it was possible to determine that there was a direct relationship between COVID-19’s impact and stress -particularly due to its disengagement dimension- and an indirect relationship between both variables, mediated by inclusion with the organization. The indirect results are described in Table 8.

**Table 8:** Testing indirect effect models.

	*Direct effect*	*Indirect effect*	*Total effect*
Impact of COVID on disengagement, mediated by organizational inclusion	0.32***	0.17**	0.49**

*p < .05

**p < .01

***p < .001

In light of the conducted tests, it is tentatively plausible to conclude that the impact of COVID-19 affects stress levels in social service workers, both directly and indirectly, through their level of inclusion within their respective organizations. and considering the gender, age, ethnicity, and SES of the workers.

## Discussion

The study conducted on nonprofit human service organization workers in Chile has revealed that the impact of COVID-19 is directly associated with increased workplace stress. At the same time, the study has shown that the relationship is mediated by organizational inclusion. In other words, individuals who experienced a more significant disruption in their lives due to COVID-19 were more likely to experience heightened work-related stress. This heightened stress was a direct consequence of the pandemic’s adverse effects on their quality of life and the negative impact of COVID-19 on their perception of inclusion within the organization. In essence, the negative impact of COVID-19 on quality of life not only generated stress but also had an adverse effect on individuals’ perception of inclusion, which, in turn, was negatively correlated with their stress levels.

Following the premises of the job demands-resources theory [40], these results assume that the effect of COVID-19 on mental health, physical health, and personal safety introduced an additional demand for these workers. Consequently, they experienced elevated levels of stress, notably reflected in their disengagement from work, as evidenced by the measure developed by Demerouti [56]. These results align with previous research, such as Khodami et al. [36], which reported an increased stress associated with COVID-19, particularly as the duration of quarantine extended, confirming that the effects of COVID-19 acted as cognitive, physical, and emotional demands.

Moreover, the pandemic reshaped the dynamics of relationships, impacting their nature and frequency. Individuals were not only required to maintain distance from their workplace or adopt remote work but also had to carry out their tasks in a more solitary manner [60]. This transformation affected the traditional spaces for interaction, socialization, and support that were previously available [61].

Our study provides several noteworthy insights into the realm of inclusion. First, it underscores the pivotal role of organizational inclusion in influencing stress levels. It reaffirms the importance of inclusion in organizational outcomes, a trend corroborated by previous research. However, it was not possible to observe the effect of inclusion with the working group, higher management, or supervisors on stress. This outcome may be linked to the fact that teams and supervisors found more effective ways to adapt to the new context, while the organizational and structural dimensions posed greater challenges, thus affecting inclusion processes and stress levels.

Considering the impact of COVID-19 on stress and inclusion within organizations, it is pertinent to question how human service organizations and social services are addressing the well-being of their workers in times of crisis—a topic that has been underexplored by both organizations and academics, whether as research or a dimension for COVID-19 adaptation [2, 11]. Often, worker conditions receive less attention compared to the well-being of their clients, as if there were an inherent conflict between the welfare of workers, service quality, and organizational sustainability [62].

Research has established that in human service organizations, the quality of job relationships plays a crucial role in mitigating the impact of stress [63]. While workers who actively choose to work for social causes tend to perform better, this alone is insufficient [64]. Our study underscores the need for post-COVID interventions aimed at ensuring equal access to information, equitable participation, and decision-making.

Furthermore, it is essential to explore ways to promote inclusion and create inclusive environments amid catastrophic situations when decisions need to be efficient, actions swift, and participation diligent. As previously emphasized, leaders must commit to fostering inclusive climates, a factor shown in this sector to be associated with favorable outcomes for individuals and organizations [15]. In this context, the role of inclusive leaders who facilitate meaningful connections beyond formal roles and offer equal and equitable treatment becomes paramount [65].

In this regard, harnessing information and communication technologies to enhance social services management, particularly in extreme situations, seems crucial. However, it is important to note that, despite work organization adjustments, social services still require a substantial in-person dimension and must continue their on-site work. This necessitates the development of strategies to address the demands and stress inherent in a crisis context, characteristic of the "turn of business."

## Conclusions

The connection between the impact of COVID-19 on quality of life and stress has not been extensively explored within nonprofit organizations and social services. This study represents one of the initial investigations to propose both a direct and indirect association through the mediating factor of inclusion. In this context, our findings in the examined sample provide evidence that individuals who experienced greater stress due to the pandemic also exhibited heightened levels of workplace stress.

The study results should be treated with caution, especially given the unique conditions that social service workers in Chile contend with. These conditions include limited budgets and the introduction of new public management models, which often add administrative tasks that can become general stressors [66, 67]. Another limitation of the study is related to the measure of inclusion, which did not encompass the informal participation subscale due to quarantine restrictions. In cultures such as the Chilean, informality plays a significant role in labor relations.

Regarding practical implications, our results underscore the need for ongoing stress prevention efforts. Nonprofit organizations must take steps to reduce stress and exhaustion, as workers are particularly prone to heightened stress levels during catastrophes. This heightened stress can lead to increased levels of burnout and even attrition from organizations. It is essential to bolster mindfulness and explore job-crafting at the individual level [68, 69], strengthen social support and the role of supervision at the group level [70, 71], and ensure the review of tasks, job redesign, technology integration, information sharing, and the mechanisms of participation in decision-making at the organizational level [72].
